# Bayesian Genomic Prediction with Genotype **×** Environment Interaction Kernel Models

**DOI:** 10.1534/g3.116.035584

**Published:** 2016-10-28

**Authors:** Jaime Cuevas, José Crossa, Osval A. Montesinos-López, Juan Burgueño, Paulino Pérez-Rodríguez, Gustavo de los Campos

**Affiliations:** * Universidad de Quintana Roo, Chetumal, Quintana Roo, México; † Biometrics and Statistics Unit, International Maize and Wheat Improvement Center (CIMMYT), 06600 México D. F., México; ‡ Facultad de Telemática, Universidad de Colima, C. P. 28040, Edo. de Colima, México; § Colegio de Postgraduados, C. P. 56230 Montecillos, Edo. de México, México; ** Department of Epidemiology and Biostatistics, Michigan State University, East Lansing, Michigan 48824

**Keywords:** genomic selection, multi-environment, kernel GBLUP, Gaussian kernel, marker × environment interaction, GenPred, Shared data resource

## Abstract

The phenomenon of genotype × environment (G × E) interaction in plant breeding decreases selection accuracy, thereby negatively affecting genetic gains. Several genomic prediction models incorporating G × E have been recently developed and used in genomic selection of plant breeding programs. Genomic prediction models for assessing multi-environment G × E interaction are extensions of a single-environment model, and have advantages and limitations. In this study, we propose two multi-environment Bayesian genomic models: the first model considers genetic effects (u) that can be assessed by the Kronecker product of variance–covariance matrices of genetic correlations between environments and genomic kernels through markers under two linear kernel methods, linear (genomic best linear unbiased predictors, GBLUP) and Gaussian (Gaussian kernel, GK). The other model has the same genetic component as the first model (u) plus an extra component, ***f***, that captures random effects between environments that were not captured by the random effects u. We used five CIMMYT data sets (one maize and four wheat) that were previously used in different studies. Results show that models with G × E always have superior prediction ability than single-environment models, and the higher prediction ability of multi-environment models with u and f over the multi-environment model with only ***u*** occurred 85% of the time with GBLUP and 45% of the time with GK across the five data sets. The latter result indicated that including the random effect ***f*** is still beneficial for increasing prediction ability after adjusting by the random effect u.

The long, rich history of the development of statistical models for assessing genotype × environment (G × E) interaction in agricultural and plant breeding experiments precedes the development of the analysis of variance. Fisher and Mackenzie (1923) pointed out that the differential responses of genotypes to environments could be better fitted by a product operator (multiplicative) than by a sum formula. More than a decade later, a multiplicative operator consisting of a simple linear regression of line performance on the environmental mean was proposed by Yates and Cochran (1938) (joint-regression analysis). This is a method that approximates G × E interaction by one multiplicative term. Several decades later, other multiplicative operators based on singular value decomposition (SVD) of the G × E were proposed within the framework of linear–bilinear, fixed-effect models (Cornelius *et al.* 1996). Later, Piepho (1998) and Smith *et al.* (2001, 2005) employed the SVD operator for modeling G × E but in the context of multivariate linear mixed-effect models, while Crossa *et al.* (2004, 2006) and Burgueño *et al.* (2008) considered using structured covariance matrices to model G × E based on pedigree linear mixed models for estimating the BLUP of the breeding values. These models account for the average performance of the interaction across the entire genome without distinguishing parts of the genome that may be more influenced by the environment than others, and using environments without characterizing climatic factors that may interact with regions of the genome.

The first to propose whole-genome regression methods (genomic selection, GS) by jointly fitting hundreds of thousands of markers with major as well as small effects were Meuwissen *et al.* (2001). Implementing whole-genome regression methods poses important statistical and computational challenges because the number of markers (*p*) greatly exceeds the number of data points (*n*) available; however, shrinkage estimation procedures allow the implementation of whole-genome regression methods. Recently, standard GS models were extended to multi-environment data. For instance, Burgueño *et al.* (2012) were the first to use a multi-environment version of the GBLUP where G × E was modeled using genetic correlations; however, they did not attempt to incorporate environmental variables as surrogates for environments. Jarquín *et al.* (2014) proposed a Bayesian reaction norm model that is a type of random effects model where the main effects of markers and environmental covariates (ECs), as well as the interactions between markers and ECs, are introduced using covariance structures that are functions of marker genotypes and ECs. The proposed approach represents an extension of the GBLUP and can be interpreted as a random effects model on all the markers, all the ECs, and all the interactions between markers and ECs using a multiplicative operator.

The reaction norm model of Jarquín *et al.* (2014) has some limitations, for example, the Gaussian prior does not induce variable selection and the shrinkage induced by Gaussian prior density may not be particularly appropriate when markers or ECs may have large effects. Furthermore, the reaction norm model considers the case of a particular multiplicative interaction model and, as such, may be considered a simple approximation to the complex phenomenon of interaction between genes and environmental conditions which, in practice, may take many different forms.

To solve some of the challenges of the reaction norm model, López-Cruz *et al.* (2015) proposed a marker × environment interaction model where marker effects and genomic values are partitioned into components that are stable across environments (main effects) and others that are environment-specific (interactions); this interaction model is useful when selecting for stability and adaptation to target environments. Consistently, genomic prediction ability increased substantially when incorporating G × E or marker × environment interaction. The marker × environment interaction model has some advantages over previous models: it is easy to implement in standard software for GS and can be implemented with any Bayesian priors commonly used in GS, including not only shrinkage methods (*e.g.*, GBLUP), but also variable selection methods (which cannot be directly implemented under the reaction norm model) (Crossa *et al.* 2016).

The marker × environment interaction model of López-Cruz *et al.* (2015) estimates the phenotypic correlation between any two environments as a ratio of variance components, thus forcing the covariance between pairs of environments to be positive. Therefore, the marker × environment interaction model is appropriate for use with sets of environments that are positively correlated. However, in practice, this G × E pattern may be too restrictive in cases where several environments have close to zero correlations; this determines a large variance component of G × E as compared with the genetic variance component (Burgueño *et al.* 2011).

In a recent article, Cuevas *et al.* (2016) applied the marker × environment interaction GS model of López-Cruz *et al.* (2015), but modeled not only through the standard linear kernel (GBLUP), but also through a nonlinear GK similar to that used in the Reproducing Kernel Hilbert Space with Kernel Averaging (RKHS KA) (de los Campos *et al.* 2010) and with the bandwidth estimated using an empirical Bayesian method (Pérez-Elizalde *et al.* 2015). The methods proposed by Cuevas *et al.* (2016) were used to perform single-environment analyses and extended to account for G × E interaction in wheat and maize data sets. In single-environment analyses, the GK had higher prediction ability than GBLUP for all environments. For cross-validation where some lines are observed only in some environments and predicted in others, the multi-environment G × E interaction model with GK resulted in prediction accuracies up to 17% higher than that of the multi-environment G × E interaction model with GBLUP linear kernel. For the maize data set, the prediction ability of the multi-environment model with GK was on average 5–6% higher than that of the multi-environment GBLUP. Cuevas *et al.* (2016) concluded that the higher prediction ability of the GK models coupled with the G × E model is due to more flexible kernels that account for small, more complex marker main effects and marker-specific interaction effects. However, the marker × environment interaction model using the GK of Cuevas *et al.* (2016) also assumes sets of environments that are positively correlated (as in López-Cruz *et al.* 2015).

In this study, we propose two multi-environment G × E genomic models that attempt to overcome some of the restrictions of previous genomic models. The main objective was to compare the prediction ability of the two proposed multi-environment G × E genomic models, each used with two kernel methods: linear (GBLUP) and nonlinear (GK). One multi-environment G × E model considers the genetic effects ***u*** that is modeled by the Kronecker product of the variance–covariance matrix of genetic correlations between environments with the genomic relationship between lines (using GBLUP or GK methods); this model with ***u*** is parsimonious because it estimates the combination of the genetic main effect plus the unstructured genetic variance–covariance interaction matrix between environments. The other model has the same genetic components as the previous one (u) plus an extra component, ***f***, that attempts to capture random effects between environments that were not captured by u. Both genomic prediction models assume that errors have a diagonal variance–covariance matrix (Σ).

A total of five extensive genomic data sets were used to compare the prediction ability of the two multi-environment G × E genomic models (each with GBLUP and GK methods) among themselves and with the prediction ability obtained by the single-environment (also with GBLUP and GK methods). These five data sets have been used in previous genomic studies where prediction ability was assessed only for individuals observed in some environments but not in others (cross-validation method 2, CV2, by Burgueño *et al.* 2012).

## Methods

### Statistical models

#### Single-environment model:

The semiparametric regression model for each single environment (j=1,…,m environments) of lines $\left(i=1,\ldots ,n_{j}\right)$ is given by:

$$y_{j}=1_{n_{j}}\mu_{j}+u_{j}+\varepsilon_{j}$$ (1)

where $y_{j}$ is the response vector containing $n_{j}$ phenotypic values, $1_{n_{j}}$ is a vector of ones of order $n_{j},$ $\mu_{j}$ is the overall mean of the $j^{th}$ environment, and the random vectors of the genetic values $u_{j}$ and the errors $\varepsilon_{j}$ are independent random variables with $u_{j}∼N\left(0,\;\sigma_{u_{j}}^{2}K_{j}\right)$ and $\varepsilon_{j}∼N\left(0,\;\sigma_{\varepsilon_{j}}^{2}I_{n_{j}}\right),$ respectively, where $\sigma_{u_{j}}^{2}K_{j}$ is the variance of $u_{j},$ $K_{j}$ is a symmetric semipositive definite matrix representing the covariance of the genetic values, and $\varepsilon_{j}$ is the vector of random errors in the $j^{th}$ environment with normal distribution and common variance $\sigma_{\varepsilon_{j}}^{2}.$ The biallelic p centered and standardized molecular markers in the $j^{th}$ environment are represented in incidence matrix $X_{j}$ such that $K_{j}=G_{j}=\left(X_{j}X_{j}^{\prime }/p\right)$ is a linear kernel. Model (1) is known as the GBLUP (VanRaden, 2007, 2008). Single-environment model (1) is similar to model (1.2) of Pérez-Elizalde (2015) when the linear kernel is used.

It should be noted that under the conditions given above, model (1) estimates the genomic relationship by means of its linear kernel $\left(X_{j}X_{j}^{\prime }\right).$ However, nonlinear kernels such as the GK can also be used (Cuevas *et al.* 2016). The GK commonly used in genomic prediction is $K_{j}\left(x_{ji},x_{ji^{\prime }}\right)=\exp\left(-h_{j}d_{ii^{\prime }}^{2}\right)$ (Pérez-Rodríguez *et al.* 2012), where $d_{ii^{\prime }}$ is the distance based on markers between individuals $i,i^{\prime }$ $\left(i=1,\ldots ,n_{j}\right)$ for the $j^{th}$ environment and $h_{j}>0$ is a bandwidth parameter, which in this study is estimated based on the Bayesian method proposed by Pérez-Elizalde *et al.* (2015).

#### Multi-environment models:

For multi-environments, the random model considers that the individuals between environments are correlated such that there is a genetic correlation between environments that can be modeled with matrices of order m×m. Therefore, the extension of random model (1) that accounts for genetically correlated environments is expressed as

y=μ+u+ε  (2)

where $y=[\begin{array}{ccccc} y_{1} & \cdots & y_{j} & \cdots & y_{m} \end{array}]\prime ;$ $\mu =[\begin{array}{ccccc} 1_{n_{1}}\mu_{1} & \cdots & 1_{n_{j}}\mu_{j} & \cdots & 1_{n_{m}}\mu_{m} \end{array}]\prime ;$ $u=[\begin{array}{ccccc} u_{1} & \cdots & u_{j} & \cdots & u_{m} \end{array}]\prime ;$ and $\varepsilon =[\begin{array}{ccccc} \varepsilon_{1} & \cdots & \varepsilon_{j} & \cdots & \varepsilon_{m} \end{array}]\prime$ where μ is the vector with the intercept of each environment, and random vectors u, and ε are independent and normally distributed (Burgueño *et al.* 2012) with $u∼N\left(0,K_{0}\right)$ and ε∼N(0,R).

When the number of individuals included in each environment is different, then

$$K_{0}=\left[\begin{array}{ccccc} \sigma_{u_{1}}^{2}K_{1} & \ldots & \sigma_{u_{1}u_{j}}K_{1j} & \;\ldots & \sigma_{u_{1}u_{m}}K_{1m}\;\\ \vdots & \ddots & \vdots & \ddots & \vdots \\ \sigma_{u_{j}u_{1}}K_{j1} & \ldots & \sigma_{u_{j}}^{2}K_{j} & \ldots & \;\sigma_{u_{j}u_{m}}K_{jm}\;\\ \vdots & \ddots & \;\vdots & \ddots & \vdots \\ \sigma_{u_{m}u_{1}}K_{m1} & \ldots & \sigma_{u_{m}u_{j}}K_{mj} & \ldots & \sigma_{u_{m}}^{2}K_{m} \end{array}\right]$$

where $\sigma_{u_{j}}^{2}$ is the genetic variance of the *j*^th^ environment and $\sigma_{u_{j}u_{j}^{\prime }}$ is the genetic covariance between two environments, *j*^th^ and *j*^th’^, $K_{j}$ is the kernel constructed with the markers of the individuals in the *j*^th^ environment and $K_{jj^{\prime }}$ is the kernel constructed with the markers of the individuals included in the two environments, *j*^th^ and *j*^th’^. Also, the residual matrix is assumed to be diagonal

$$R=\left[\;\begin{array}{ccccc} \sigma_{\varepsilon_{1}}^{2}I_{n_{1}} & \ldots & 0 & \;\ldots & 0\\ \vdots & \ddots & \vdots & \ddots & \vdots \\ 0 & \ldots & \sigma_{\varepsilon_{j}}^{2}I_{n_{j}} & \ldots & 0\;\\ \vdots & \ddots & \;\vdots & \ddots & \vdots \\ 0 & \ldots & 0 & \ldots & \sigma_{\varepsilon_{m}}^{2}I_{n_{m}} \end{array}\right]$$

where $\sigma_{\varepsilon_{j}}^{2}$ is the random error of the environment.

When the number of individuals in the environments is the same $\left(n_{j}=n_{j^{’}}=n\right)$, the kernels are the same $K_{j}=K,$ and the identity matrices are the same $I_{j}=I$, then $K_{0}=U_{E}⊗K,$ and R=Σ⊗I, where ⊗ denotes the Kronecker product and ***K*** is unique (calculated for all genotypes, regardless of the environment in which they were tested) and could be the genomic relationship matrix as defined for model (1). The matrix $K_{0}$ is the product of one kernel with information between environments $\left(U_{E}\right)$ and another kernel with information between lines based on markers (K), similar to the multi-task Gaussian process (Bonilla *et al.* 2007). The mixed model used in genomic prediction can have several structures for modeling matrix $U_{E}\;$ (Burgueño *et al.* 2012). When there are not many environments, the unstructured variance–covariance could be used for $U_{E},\;$of order m×m such that

$$U_{E}=\left[\;\begin{array}{ccccc} \sigma_{u_{1}}^{2} & \ldots & \sigma_{u_{1}u_{j}} & \ldots & \sigma_{u_{1}u_{m}}\;\\ \vdots & \ddots & \vdots & \ddots & \vdots \\ \sigma_{u_{j}u_{1}} & \ldots & \sigma_{u_{j}}^{2} & \ldots & \;\sigma_{u_{j}u_{m}}\;\\ \vdots & \ddots & \vdots & \ddots & \vdots \\ \sigma_{u_{m}u_{1}} & \ldots & \sigma_{u_{m}u_{j}} & \ldots & \sigma_{u_{m}}^{2} \end{array}\right]$$

where the *j^th^* diagonal element of the m×m matrix $U_{E}$ is the genetic variance $\sigma_{u_{j}}^{2}$ within the *j^th^* environment, and the off-diagonal element is the genetic covariance $\;\sigma_{u_{j}u_{j^{\prime }}}$ between the *j^th^* and *j^th^*^’^ environments. For a large number of environments, a factor analytical model usually performs as well or better than the unstructured model (Burgueño *et al.* 2012). Also, matrix **Σ** is an error diagonal matrix of order m×m, *i.e.*, $\Sigma =\mathrm{diag}\left(\sigma_{\varepsilon_{1}}^{2},\ldots .,\sigma_{\varepsilon_{m}}^{2}\right).$

As described, model (2) is parsimonious because it expresses the genetic values within the environment derived from the markers plus the interaction between these genetic values with the environments. Model (2) can be used with the linear kernel matrix **G** or with the GK that allows capturing small cryptic genetic epistatic effects.

Jarquín *et al.* (2014) argued that due to “imperfect linkage disequilibrium (LD) between markers and genes at causal loci or because of model misspecification (*e.g.*, interactions between alleles that are unaccounted for), the regression on markers may not fully describe genetic differences among lines.” Therefore, it is reasonable to add another component that would attempt to model the variation between individuals that was not captured by u. Thus, we added to model (2) a random component ***f*** representing the genetic variability among individuals that was not accounted for as a function of the markers in component u.

Therefore, multi-environments with random effects considering genetic correlations between environments (model (2)) can be extended by adding an extra variability to account for the genetic variance among individuals across environments that was not explained by u, that is ***f***. Therefore, the extension of the random linear model (2) is expressed as

y=μ+u+f+ε  (3)

where $f=[\begin{array}{ccccc} f_{1} & \cdots & f_{j} & \cdots & f_{m} \end{array}]\prime$ with the random vectors f independent of u and normally distributed f∼N(0,Q). In general, when the number of individuals is not the same in all environments,

$$Q=\left[\begin{array}{ccccc} \sigma_{f_{1}}^{2}I_{n_{1}} & \ldots & \sigma_{f_{1}f_{j}}I_{n_{1}} & \;\ldots & \sigma_{f_{1}f_{m}}I_{n_{1}}\;\\ \vdots & \ddots & \vdots & \ddots & \vdots \\ \sigma_{f_{j}f_{1}}I_{n_{j}} & \ldots & \sigma_{f_{j}}^{2}I_{n_{j}} & \ldots & \;\sigma_{f_{j}f_{m}}I_{n_{j}}\;\\ \vdots & \ddots & \vdots & \ddots & \vdots \\ \sigma_{f_{m}f_{1}}I_{n_{m}} & \ldots & \sigma_{f_{m}f_{j}}I_{n_{m}} & \ldots & \sigma_{f_{m}}^{2}I_{n_{m}} \end{array}\right]$$

where $\sigma_{f_{j}}^{2}$ is the genetic effects in the *j*^th^ environment not explained by the random genetic effect u and $\sigma_{f_{j}f_{j^{\prime }}}$ is the covariance of the genetic effects between two environments not explained u. When the number of individuals is the same in all the environments, then $Q=F_{E}⊗I.$

Matrix $F_{E},$ is unstructured and captures genetic variance–covariance effects between the individuals across environments that were not captured by the $U_{E}$ matrix; in this case, matrix $F_{E}$ can be expressed as

$$F_{E}=\left[\begin{array}{ccccc} \sigma_{f_{1}}^{2} & \ldots & \sigma_{f_{1}f_{j}} & \ldots & \sigma_{f_{1}f_{m}}\;\\ \vdots & \ddots & \vdots & \ddots & \vdots \\ \sigma_{f_{j}f_{1}} & \ldots & \sigma_{f_{j}}^{2} & \ldots & \sigma_{f_{j}f_{m}}\\ \vdots & \ddots & \vdots & \ddots & \vdots \\ \sigma_{f_{m}f_{1}} & \ldots & \sigma_{f_{m}f_{j}} & \ldots & \sigma_{f_{m}}^{2} \end{array}\right]$$

where the *j^th^* diagonal element of the m×m matrix $F_{E}$ is the genetic environmental variance $\sigma_{f_{j}}^{2}$ within the *j^th^* environment, and the off-diagonal element is the genetic covariance $\sigma_{f_{j}f_{j^{\prime }}}$ between environments *j* and *j*’.

#### Considerations on the application of the proposed models:

An objective of this article was to compare the use of linear and nonlinear kernels for matrix ***K*** to determine the relationship between lines, for each of the three models described earlier. Thus, for each of the five data sets, we fitted models (1)–(3) for ***K*** as a linear kernel using the GBLUP, and ***K*** as a nonlinear GK with the bandwidth parameter estimated according to Pérez-Elizalde *et al.* (2015) on model (1).

The same numbers of individuals in each environment were employed in these applications. Therefore, in this case, the same kernel ***K*** constructed for all individuals was developed. Also, the observations in each environment were standardized with the aim of examining and comparing the proportion of variance components explained by each random component of the single-environment model with those from the two multi-environment models, and also between the variance components of the random effects ***u*** and ***f*** of models (2) and (3). Although the intercepts were expected to be close to zero, they were included in the model as parameters to be estimated.

#### Implementation of Bayesian models:

Single-environment model (1) was fitted with the Bayesian Generalized Linear Regression (BGLR) package of de los Campos and Pérez-Rodríguez (2014). The BGLR considers a Bayesian model and, from that point of view, a linear mixed model is a three-stage hierarchical model (Jiang 2007). In the first stage, the distribution of the observations given the random effects is defined and, in the second stage, the distribution of the random effects given the model parameters is added. In the last stage, a prior distribution is assumed for the parameters. Under normal distribution these stages may be specified as follows: the conditional distribution of the data from the *j*^th^ environment is $p\left(y_{j}|\mu_{j},u_{j},\sigma_{\varepsilon_{j}}^{2}\right)=N\left(y_{j}|1_{n_{j}}\mu_{j}+u_{j},\sigma_{\varepsilon_{j}}^{2}I\right);$ in the second stage, the conditional distribution of random effects is $p\left(u_{j}|\sigma_{u_{j}}^{2},K_{j}\right)=N\left(u_{j}|0,\sigma_{u_{j}}^{2}\;K_{j}\right);$ finally, in the last stage, it is assumed that the prior distribution can be expressed as $p\left(\mu_{j},\sigma_{\varepsilon_{j}}^{2},\sigma_{u_{j}}^{2}|S_{\varepsilon },\;df_{\varepsilon },S_{u},\;df_{u}\right)=p\left(\mu_{j}\right)p\left(\sigma_{\varepsilon_{j}}^{2}|S_{\varepsilon },df_{\varepsilon }\right)p\left(\sigma_{u_{j}}^{2}|S_{u},df_{u}\right),$ with a flat prior for $\mu_{j}$ and with a scaled inverse Chi-squared prior distribution for the error variance $p\left(\sigma_{\varepsilon_{j}}^{2}|S_{\varepsilon },df_{\varepsilon }\right)=\chi^{-2}\left(\sigma_{\varepsilon_{j}}^{2}|S_{\varepsilon },df_{\varepsilon }\right)$ with scale factor $S_{\varepsilon }$ and $df_{\varepsilon }>0$ degrees of freedom, while the prior for $\sigma_{u_{j}}^{2}$ is scaled inverse Chi-squared distribution $p\left(\sigma_{u_{j}}^{2}|S_{u},df_{u}\right)=\chi^{-2}\left(\sigma_{u_{j}}^{2}|S_{u},df_{u}\right)$ with scale factor $S_{u}$ and degrees of freedom $df_{u}>0.$

The hyperparameters were set using the rules given by Pérez-Rodríguez and de los Campos (2014). In this study, we assumed default values of $df_{\varepsilon }=df_{u}=5,$ with the intention of avoiding infinite variance values. We also assumed that the model explained 50% of the phenotypic variance; then $S_{\varepsilon }=0.5\mathrm{var}\left(y_{j}\right)\left(df_{\varepsilon }+2\right),$ $S_{u}=0.5\mathrm{var}\left(y_{j}\right)\left(df_{u}+2\right)/\text{mean}\left(\text{diag}\left(K_{j}\right)\right).$ More details on the use of the BGLR can be found in Pérez-Rodríguez and de los Campos (2014).

Multi-environment models (2) and (3) were fitted using the Multi-Trait Model (MTM) software of de los Campos and Grüneberg (2016) that uses a Bayesian approach, assuming the $K_{j}$ are the same in all the environments and considering that, at the first level, the conditional distribution of the data can be modeled by a multivariate normal distribution p(y|μ,u,f,Σ)=N(y|μ+u+f,Σ⊗I). At the second level, the prior distributions for u and f are multivariate normal with mean vector zero and variance–covariance matrices $U_{E}⊗K,$ and $F_{E}⊗I,$ respectively, that is, $p\left(u|U_{E},K\right)=N\left(u|0,U_{E}⊗K\right),$ $p\left(f|F_{E}\right)=N\left(f|0,F_{E}⊗I\right).$ At the third level, a flat prior distribution for the intercepts of each environment is used, and the prior distributions of $U_{E}$ and $F_{E}$ are inverse Whishart $p\left(U_{E}|S_{0},df_{0}\right)=p\left(F_{E}|S_{0},df_{0}\right)=W^{-1}\left(S_{0},df_{0}\right),$ where the scale matrix $S_{0}$ is an identity matrix of order *m* (number of environments) and the degrees of freedom $df_{0}=m.$ For the prior distribution of the elements of $\sigma_{\varepsilon_{j}}^{2}$ of the diagonal of Σ, we used a scaled inverse Chi-squared distribution with the hyperparameters’ degree of freedom and a scaled factor equal to 1.

### Software

Both packages, BGLR and MTM, fit the models with Markov Chain Monte Carlo (MCMC) using the Gibbs sampler with 30,000 iterations, with a burn-in of 5000 and a thinning of five, so that 5000 samples were used for inference. Convergence and diagnostic tests were performed. The Gelman-Rubin convergence tests for all parameters of the three models were satisfactory, using lag-5 thinning results in low autocorrelations in each of the three models. The Raftery–Lewis test suggested a small burn-in between 10,000 and 20,000 iterations for the five data sets used.

The *R* codes with a brief description for fitting multi-environment model (3) using the MTM package of de los Campos and Grüneberg (2016) are given in Appendix A.

### Assessing prediction ability

Prediction ability was assessed using 50 TRN-TST (TRN = training and TST = testing) random partitions; we used this approach because it provides higher precision in the predictive estimates than the framework that uses different numbers of folds. For single-environment model (1), 50 random partitions were formed with 70% of the observations in the training set and 30% of the observations in the testing set.

For multi-environment models (2) and (3), we simulated the prediction problem that assumes that 70% of the individuals were observed in some environments but not in others (CV2, Burgueño *et al.*, 2012). We used the procedure of López-Cruz *et al.* (2015) to assign individuals to the training and testing sets. We formed TRN sets with 70% of the n ×m observations and TST sets with 30% of the n ×m observations to be predicted (their phenotypic values were not observed and appear as missing).

In each random partition, Pearson’s correlations between the predicted and observed values for each environment were computed; these are considered the prediction accuracies of those models, and thus the average correlation for all random partitions and their standard deviation are reported. The variance components of the three models using the full data are also reported.

When random cross-validation partitions simulated the prediction of a portion of individuals that represents newly developed lines not observed in any environment (random cross-validation 1, CV1, Burgueño *et al.* 2012), it is possible that ***f*** (of model (3)) could account for part of the random error. However, in this study, we observed all the individuals in at least one environment and predicted other individuals that were not observed in some environments (random CV2, Burgueño *et al.* 2012); therefore, under CV2 random cross-validation, ***f*** is predictable.

### Experimental data sets

In this study, we used five data sets that have been used in different studies. Wheat data set 1 was used by Crossa *et al.* (2010) and Cuevas *et al.* (2016), maize data set 2 was employed in the studies of Crossa *et al.* (2013) and Cuevas *et al.* (2016), and wheat data sets 3–5 were analyzed by López-Cruz *et al.* (2015). Brief descriptions of the phenotypic and marker data sets are given below.

#### Wheat data set 1:

This data set, from CIMMYT’s Global Wheat Program, was used by Crossa *et al.* (2010) and Cuevas *et al.* (2016) and includes 599 wheat lines derived from 25 yr (1979–2005) of Elite Spring Wheat Yield Trials (ESWYT). The environments represented in these trials were grouped into four basic agroclimatic regions (mega-environments). The phenotypic trait considered here was grain yield (GY) of the 599 wheat lines evaluated in each of the four mega-environments. The 599 wheat lines were genotyped using 1447 Diversity Array Technology (DArT) markers generated by Triticarte Pty. Ltd. (Canberra, Australia; http://www.triticarte.com.au). Markers with a minor allele frequency (MAF) < 0.05 were removed, and missing genotypes were imputed using samples from the marginal distribution of marker genotypes. The number of DArT markers after edition was 1279.

#### Maize data set 2:

This data set was first used by Crossa *et al.* (2013) and then by Cuevas *et al.* (2016); it includes a total of 504 double-haploid (DH) maize lines obtained by crossing and backcrossing eight parents that formed 10 full-sib (backcrosses) and six sib families. Each DH line was crossed to an elite single-cross hybrid of the opposite heterotic group to produce 504 testcrosses. The trait analyzed in this study was GY (kg/hectare) in three optimum rain-fed trials. The field experimental design in each of the three environments was an α-lattice incomplete block design with two replicates. Data were preadjusted using estimates of incomplete blocks nested in replicates.

The initial total of 681,257 genotyping-by-sequencing (GBS) markers had a percentage of missing cells per chromosome ranging from 51.3 to 52.8%; after editing, this percentage decreased to around 43–44% of the total number of cells. Around 20% of cells were missing in the edited GBS information used for prediction after imputation. After filtering markers for MAF, a total of 158,281 GBS were used for prediction.

#### Wheat data sets 3–5:

These three data sets were described and used by López-Cruz *et al.* (2015) for proposing a marker × environment interaction model. The phenotypic data consisted of adjusted GY (tonnes/hectare) records collected during three evaluation cycles of different inbred lines evaluated in different environments. All trials were established using an α-lattice design with three replicates in each environment at CIMMYT’s main wheat breeding station at Cd. Obregon, Mexico. The environments were three irrigation regimes (moderate drought stress, optimal irrigation, and drought stress), two planting systems (bed and flat planting), and two different planting dates (normal and late). The phenotype used in the analysis was the Best Linear Unbiased Estimate (BLUE) of GY obtained from a linear model applied to the α-lattice design of each cycle-environment combination.

Wheat data set 3 had 693 wheat lines evaluated in four environments, wheat data set 4 included 670 wheat lines evaluated in four environments, and wheat data set 5 had 807 wheat lines evaluated in five environments. Genotypes were derived using GBS technology, and markers with a MAF < 0.05 were removed. All markers had a high incidence of uncalled genotypes, so we applied thresholds for incidence of missing values and focused on maintaining relatively large and similar numbers of markers per data set. After editing the missing markers, we had a total of 15,744 GBS markers for analyzing wheat data sets 3 and 4, and 14,217 GBS markers available for analyzing wheat data set 5.

### Data availability

Phenotypic and marker data for the five data sets can be downloaded from http://hdl.handle.net/11529/10710.

## Results

In the following sections, we present prediction accuracies for each data set, and describe two main comparisons: (1) method GBLUP *vs.* method GK for models (1)–(3); and (2) model (1) *vs.* model (2) and model (3) *vs.* model (2) for methods GBLUP and GK.

### Wheat data set 1

Results showed increased prediction ability for models (1) and (2) for GK over GBLUP ranging from 12 to 16% for E1, E3, and E4. Also, model (3) showed 12 and 9% increases in prediction ability of GK over GBLUP for E1 and E4, respectively (Table 1), whereas the percent difference of GK model (3) *vs.* GBLUP model (3) was only −1 and 1% for E2 and E3, respectively.

**Table 1:** Percent change in prediction accuracy of GK *vs.* GBLUP for each of the three models (1)–(3), prediction accuracy of model (2) *vs.* model (1) for GK and GBLUP, and prediction accuracy of model (3) *vs.* model (2) for GK and GBLUP for each environment in each data set

Environment	GK *vs.* GBLUP			Model (2) *vs.* Model (1)		Model (3) *vs.* Model (2)	
	Model (1)	Model (2)	Model (3)	GBLUP	GK	GBLUP	GK
Wheat data set 1							
E1	15	12	12	2	0	6	5
E2	1	8	−1	34	44	13	4
E3	14	16	1	60	62	17	2
E4	14	11	9	12	9	5	3
Maize data set 2							
E1	4	7	3	8	10	3	0
E2	7	2	1	12	7	1	0
E3	12	12	10	2	2	2	0
Wheat data set 3							
E1	5	4	2	14	12	2	1
E2	3	−3	−4	14	7	1	0
E3	16	8	8	12	5	1	1
E4	4	1	1	2	−1	1	0
Wheat data set 4							
E1	2	22	2	6	27	20	1
E2	−3	13	0	25	46	14	1
E3	3	4	1	15	17	4	0
E4	−2	10	−1	23	37	11	1
Wheat data set 5							
E1	9	9	9	4	4	0	0
E2	10	13	13	3	6	0	0
E3	9	9	9	0	0	0	0
E4	15	6	4	64	52	3	0
E5	6	4	0	85	81	3	0

GBLUP, genomic best linear unbiased predictors: GK, Gaussian kernel.

Empirical phenotypic correlations of zero or negative values between E1 and all the other environments (Table 2) were found, whereas E2–E4 were positively (moderately to highly) associated among themselves. Table 2 shows the average prediction ability of each of the four environments given by the three models for linear kernel (GBLUP) and nonlinear kernel (GK). The three GK models had higher prediction ability than the corresponding three GBLUP models for E1, E3, and E4, whereas the best prediction model for E2 was GBLUP model (3). Results for this data set indicate a relatively important level of G × E, basically caused by the differential response of individuals in E1 compared with their responses in the other environments.

**Table 2:** Mean prediction accuracies for the different environments of wheat data set 1, maize data set 2, and wheat data set 3 for GBLUP and GK methods, and three models including a single-environment (model (1)) and two multi-environment models (models (2) and (3))

Environment	GBLUP			GK		
	Model (1)	Model (2)	Model (3)	Model (1)	Model (2)	Model (3)
Wheat data set 1*^a^*						
E1	0.500 (0.056)	0.512 (0.043)	0.543 (0.044)	0.577 (0.043)	0.575 (0.036)	**0.606** (0.037)
E2	0.474 (0.048)	0.635 (0.042)	**0.720** (0.031)	0.477 (0.056)	0.685 (0.030)	0.713 (0.029)
E3	0.370 (0.056)	0.592 (0.045)	0.694 (0.031)	0.422 (0.053)	0.685 (0.030)	**0.699** (0.028)
E4	0.447 (0.047)	0.501 (0.040)	0.525 (0.034)	0.511 (0.044)	0.555 (0.044)	**0.572** (0.040)

Maize data set 2*^b^*						
E1	0.558 (0.038)	0.603 (0.043)	0.624 (0.045)	0.583 (0.042)	0.644 (0.037)	**0.645** (0.037)
E2	0.507 (0.049)	0.567 (0.055)	0.575 (0.054)	0.542 (0.056)	0.581 (0.057)	**0.582** (0.057)
E3	0.508 (0.051)	0.517 (0.045)	0.525 (0.046)	0.568 (0.044)	0.577 (0.044)	**0.578** (0.044)

Wheat data set 3*^c^*						
E1	0.529 (0.044)	0.603 (0.033)	0.617 (0.031)	0.557 (0.040)	0.625 (0.033)	**0.631** (0.035)
E2	0.622 (0.045)	0.706 (0.031)	**0.716** (0.029)	0.642 (0.030)	0.688 (0.033)	0.689 (0.034)
E3	0.452 (0.051)	0.506 (0.045)	0.512 (0.043)	0.523 (0.048)	0.547 (0.041)	**0.551** (0.041)
E4	0.493 (0.046)	0.504 (0.041)	0.507 (0.039)	**0.511** (0.042)	0.508 (0.053)	0.510 (0.052)


SDs are given in parentheses. The highest prediction accuracies for each environment in each data set are shown in boldface. GBLUP, genomic best linear unbiased predictors: GK, Gaussian kernel.

a Empirical phenotypic correlation between environments: E1 *vs.* E2= −0.019; E1 *vs.* E3= −0.19; E1 *vs.* E4= −0.12; E2 *vs.* E3 = 0.661; E2 *vs.* E4 = 0.411; E3 *vs.* E4 = 0.388.

b Empirical phenotypic correlation between environments: E1 *vs.* E2 = 0.388; E1 *vs.* E3 = 0.262; E 2 *vs.* E3 = 0.153.

c Empirical phenotypic correlation between environments: E1 *vs.* E2 = 0.527; E1 *vs.* E3 = 0.253; E1 *vs.* E4 = 0.259; E2 *vs.* E3 = 0.340; E2 *vs.* E4 = 0.328; E3 *vs.* E4 = 0.22.

Differences in the prediction ability of GBLUP model (2) and GBLUP model (1) were 2, 34, 60, and 12% for E1–E4, respectively; for GK these differences were 0, 44, 62, and 9% for E1–E4, respectively (Table 1). GBLUP model (3) was 6, 13, 17, and 5% more accurate than GBLUP model (2) for E1–E4, respectively. GK model (3) was 5, 4, 2, and 3% more accurate than GK model (2) for E1–E4, respectively. In summary, for all environments in wheat data set 1, model (3) had higher prediction ability than models (2) and (1) for GK and GBLUP. As for the methods, GK was better than GBLUP for all three models and environments, except E2.

Variance within environments (diagonal) and covariances between environments (off diagonal) were higher for ***f*** **(**expressed in $F_{E}$) for GBLUP than for GK (Appendix Table B1), except for cases involving E1; however, the opposite is true for ***u*** **(**expressed in $U_{E}$), where the absolute variance–covariance values and correlations for GK were larger than those for GBLUP, and therefore reflected in the increases in the prediction ability of the models and methods. Also, diagonal residuals estimates were smaller in GK than in GBLUP.

### Maize data set 2

Higher prediction ability of GK over GBLUP for models (1)–(3) ranged from 1 to 12% for E1–E3; the advantage of GK over GBLUP was lower in model (3) (3, 1, and 10% for E1–E3, respectively) than the advantage of model (2) *vs.* model (1) (Table 1). Comparing model (2) *vs.* model (1), the differences were similar for GBLUP and GK (8, 12, and 2% for GBLUP and 10, 7, and 2% for GK). There were no differences between model (3) and model (2) for GK and only small differences for GBLUP (3, 1, and 2% for E1–E3, respectively). Appendix Table B2 shows that the covariance between environments in $F_{E}$ was close to zero due to the low contribution of random component ***f*** for both the GK and GBLUP methods.

The empirical phenotypic correlations between the three environments in the maize data set showed moderate positive values (Table 2), with all the elements of covariance in matrices $U_{E}\;\text{and}\;F_{E}$ being positive or zero (Appendix Table B2). The elements of $U_{E},$ for the GK method were all larger than those for the GBLUP method, and the opposite occurred with the elements of variance–covariance matrix $F_{E}$ for GBLUP *vs.* GK and the diagonal values of residual matrix Σ (Appendix Table B2). The low values of $F_{E}$ for the GK methods produced a small increase in prediction ability of model (3) over model (2) (Table 2), although GK model (3) always gave the best predictors for the three environments (E1 = 0.645, E2 = 0.582, and E3 = 0.578) and was slightly superior to GK model (2). In general, results for this data set indicate that the prediction ability of the GK method was always superior to that of the GBLUP method, and model (3) was slightly better than model (2) and clearly superior to model (1).

### Wheat data set 3

GK models (1)–(3) were better predictors than GBLUP models (1)–(3) for the four environments except for E2 (GBLUP model (3)) and E4 (GK model (1)) (Table 2). For E1 and E2, the prediction ability of model (2) over model (1) was about 14% higher, whereas for E3 it was 12% and for E4 it was 2% (Table 1). An almost negligible increase in prediction ability (2) was observed when comparing model (3) *vs.* model (2) for GBLUP and GK for predicting individuals in all the environments (Table 1 and Table 2).

GK model (3) was the best predictor of E1 and E3, GBLUP model (3) was the best predictor of E2, and single-environment GK model (1) was the best predictor of E4 (Table 2). Similar to the results for maize data set 2, the very low values of the elements of matrix $F_{E}$ (Appendix Table B3) for the GK and GBLUP methods produced modest to negligible increases in prediction ability of GK model (3) and GBLUP model (3) over GK model (2) and GBLUP model (2) (from 0 to 2%, as indicated in the last two columns of Table 1).

### Wheat data set 4

The four environments included in this set of trials had a relatively low empirical phenotypic correlation (especially E1 *vs.* E3, with −0.054), except E2 and E4 (0.414) (Table 3). This indicates a relatively important level of G × E and therefore increases in prediction ability when modeling interaction, especially when comparing single-environment model (1) *vs.* multi-environment models (2) and (3) for the GK and GBLUP methods. For GBLUP, model (2) was a better predictor than model (1) for E1 (0.03 increase), E2 (0.10 increase), E3 (0.078 increase), and for E4 (0.100 increase), with an average increase of 17% (6, 25, 15, and 23%, respectively, Table 1). This superiority of model (2) over model (1) for all environments increased further in the GK, where it gave an average increase in prediction ability of 32% over the GBLUP (27, 46, 17, and 37%, respectively) (Table 1).

**Table 3:** Mean prediction accuracies for the different environments of wheat data sets 4 and 5 for GBLUP and GK methods, and three models including a single-environment (model (1)) and two multi-environment models (models (2) and (3))

	GBLUP			GK		
Environment	Model (1)	Model (2)	Model (3)	Model (1)	Model (2)	Model (3)
Wheat data set 4*^a^*						
E1	0.473 (0.052)	0.501 (0.041)	0.601 (0.033)	0.482 (0.040)	0.612 (0.041)	**0.616** (0.042)
E2	0.414 (0.063)	0.517 (0.049)	**0.588** (0.041)	0.401 (0.051)	0.584 (0.047)	0.587 (0.044)
E3	0.510 (0.052)	0.588 (0.044)	**0.609** (0.044)	0.524 (0.039)	**0.613** (0.038)	**0.613** (0.039)
E4	0.448 (0.054)	0.550 (0.037)	**0.611** (0.043)	0.440 (0.045)	0.603 (0.045)	0.607 (0.044)

Wheat data set 5*^b^*						
E1	0.561 (0.035)	0.585 (0.036)	0.583 (0.036)	0.614 (0.038)	**0.637** (0.032)	**0.637** (0.032)
E2	0.445 (0.051)	0.457 (0.040)	0.458 (0.040)	0.488 (0.046)	0.517 (0.037)	**0.518** (0.037)
E3	0.628 (0.037)	0.630 (0.027)	0.632 (0.026)	0.687 (0.026)	**0.688** (0.030)	**0.688** (0.030)
E4	0.360 (0.046)	0.592 (0.042)	0.608 (0.040)	0.415 (0.043)	**0.630** (0.037)	**0.630** (0.037)
E5	0.312 (0.055)	0.576 (0.036)	0.596 (0.035)	0.330 (0.047)	**0.597** (0.038)	**0.597** (0.038)


SDs are given in parentheses. The highest prediction accuracies for each environment in each data set are shown in boldface. GBLUP, genomic best linear unbiased predictors: GK, Gaussian kernel.

a Empirical phenotypic correlation between environments: E1 *vs.* E2 = 0.342; E1 *vs.* E3= –0.054; E1 *vs.* E4 = 0.311; E2 *vs.* E3 = 0.328; E2 *vs.* E4 = 0.414; E3 *vs.* E4 = 0.223.

b Empirical phenotypic correlation between environments: E1 *vs.* E2 = 0.166; E1 *vs.* E3 = 0.30; E1 *vs.* E4= –0.10; E1 *vs.* E5= –0.010; E2 *vs.* E3= –0.033; E2 *vs.* E4 = 0.122; E2 *vs.* E5 = 0.035; E3 *vs.* E4= –0.091; E3 *vs.* E5 = 0.023; E4 *vs.* E5 = 0.546.

The increase in prediction ability of model (3) over model (2) was important for GBLUP but not for GK; overall, GBLUP gave an average increase in prediction ability of model (3) over model (2) of 12%, whereas for GK this increase was, on average, 0.75% (Table 1). Although the increase in prediction ability of model (3) over model (2) in GK was marginal, overall, GK model (3) was slightly superior to GBLUP model (3) for E1 (0.601 *vs.* 0.616; Table 3) and E3 (0.609 *vs.* 0.613; Table 3); GBLUP model (3) was slightly better than GK model (3) for predicting E4 (0.611 *vs.* 0.607; Table 4) and they had similar accuracy for predicting E2 (0.588 *vs.* 0.587).

**Table 4:** Comparison of prediction accuracy of multi-environment GBLUP and GK model (3) with various other models published in refereed journals for the five data sets utilized in this study

	GBLUP		GK	
Wheat Data Set 1	FA	Model (3)	EB-G × E	Model (3)
E1	0.553	0.543	0.458	**0.606**
E2	0.611	**0.720**	0.644	0.713
E3	0.585	**0.694**	0.586	**0.694**
E4	0.51	0.525	0.543	**0.572**
	GBLUP		GK	
Maize Data Set 2	EB-G × E	Model (2)	EB-G × E	Model (3)
E1	0.618	0.624	0.630	**0.645**
E2	0.547	0.575	0.566	**0.582**
E3	0.519	0.525	0.556	**0.578**
	GBLUP		GK	
Wheat Data Set 3	GBLUP-ME	Model (3)	Model (3)	
E1	0.591	0.617	**0.631**	
E2	0.697	**0.716**	0.689	
E3	0.505	0.512	**0.551**	
E4	**0.516**	0.507	0.51	
	GBLUP		GK	
Wheat Data Set 4	GBLUP-ME	Model (3)	Model (3)	
E1	0.513	0.601	**0.616**	
E2	0.536	**0.588**	0.587	
E3	0.531	0.609	**0.613**	
E4	0.561	**0.611**	0.607	
	GBLUP		GK	
Wheat Data Set 5	GBLUP-ME		Model (3)	
E1	0.575	0.583	**0.637**	
E2	0.466	0.458	**0.518**	
E3	0.629	0.632	**0.688**	
E4	0.402	0.608	**0.630**	
E5	0.376	0.596	**0.597**	

FA (Factor Analytic) model, Burgueño *et al.* (2012); EB (Empirical Bayes)-G × E, Cuevas *et al.* (2016); GBLUP-ME, López-Cruz *et al.* (2015); The highest correlations in each row are in boldface. GBLUP, genomic best linear unbiased predictors: GK, Gaussian kernel.

Appendix Table B4 shows that GBLUP model (3) could not capture sufficient variability associated with random component ***u*** reflected in the within (diagonal) and between environment (off diagonal) variability of $\left(U_{E}\right).$ Therefore, GBLUP model (3) with ***f*** explained more of this variability, and this is reflected in the better prediction ability of GBLUP model (3) for E2 and E4. In contrast, GK model (3) explained most of the within and between environmental variance reflected in the large values of the elements of $U_{E}.$ Therefore, $F_{E}$ could not explain much; thus, the predictions of GK model (3) are similar to (although slightly lower than) those of GK model (2).

### Wheat data set 5

The gains in prediction ability of GK over GBLUP were consistent across models, ranging from 0 to 13% (Table 1). For the GBLUP method, the gains in prediction ability of model (2) over model (1) were very modest (for E1–E3), except for E4 and E5 (with high empirical phenotypic correlations of 0.546); gains in prediction ability of model (3) over model (2) were almost negligible, except for E4 and E5 (3%, Table 1).

The superior prediction ability of the three GK models over the GBLUP models is clearly shown in Table 3. Also, in contrast to GBLUP, the better prediction ability of GK model (2) over GK model (1) is clear for all the environments; interestingly, this increase in prediction ability due to adding interaction matrix $U_{E}$ to model (2) with respect to model (1) was not reflected when adding the extra variance–covariance matrix $F_{E}$ to model (3) with respect to model (2) (Appendix Table B5).

## Discussion

GBLUP and GK models (1)–(3), which were proposed, described and used in this study, are flexible and can be used not only with genomic information but also with pedigree information. We performed preliminary analyses with models (1)–(3) on wheat data set 1 using only pedigree information, but the prediction ability was not higher than any of the correlations obtained using the genomic relationship matrix (data not shown).

Models (2) and (3) proposed in this paper jointly estimate the genetic and the genotype × environments interaction effects. The proposed models are more parsimonious than those that explicitly separate and estimate genotype and environments affects from their interaction. In general, models that jointly estimated the main effects of genotypes and genotype × environment are preferred to those that do it separately because researchers are interested in examining the predicted values of pure precommercial cultivars and single crosses as well as their interaction and stability with environments.

Below we discuss some of the advantages and limitations of these G × E genomic prediction models (2) and (3).

### Multi-environment model (2)

When fitting model (2), estimations of the off-diagonal values of $U_{E}$ can be positive, close to zero, zero, or negative. This is a more flexible model than the multi-environment model of López-Cruz *et al.* (2015) with a linear kernel or the multi-environment model of Cuevas *et al.* (2016) with a nonlinear GK. Both propositions impose the restriction that the correlation between environments is positive; therefore, prediction ability of lines in environments with negative or zero correlations is low.

For model (2), the correlation between any two environments from the standardized data are equal to the sum $U_{E}+\Sigma ;$ thus, when the correlations between environments are close to zero, matrix $U_{E}$ tends to be diagonal so that model (2) will fit each environment almost independently from the other environments; this will produce prediction accuracies similar to those obtained by single-environment model (1), as evidenced in our results. When the correlation between environments is positive (or negative) and intermediate to high, matrix $U_{E}$ has positive (or negative) values in its off-diagonal; this allows borrowing information from one environment to predict other environments with positive (or negative) correlations, such that the linear or nonlinear kernels will increase the prediction ability of the lines in those environments. Therefore, the diagonal of matrix $U_{E}$ influences the prediction ability in a specific environment and the off-diagonal values of matrix $U_{E}$ affect the exchange of information between environments. According to Cuevas *et al.* (2016), in general, nonlinear kernel GK had better prediction ability than linear kernel GBLUP. These results were generally found in model (2) as well, because GK explained the variance within and between environments better than GBLUP, and this is reflected in the values of matrix $U_{E}$ (Appendix Table B1, Table B2, Table B3, Table B4, and Table B5).

Table 1 shows that the differences when comparing model (2) *vs.* model (1) are close in methods GK and GBLUP (*i.e.*, maize data set 2, wheat data set 3, and wheat data set 5); model (3) with GBLUP and with GK had negligible gains in prediction ability over model (2) with GBLUP and with GK. In contrast, when there are differences in models (2) and (1) in GK and GBLUP (*i.e.*, wheat data set 1 and wheat data set 4), GBLUP model (3) substantially increases prediction ability with respect to GBLUP model (2), but this does not seem to be the case for GK.

### Multi-environment model (3)

Model (2) explained only part of the variability, whereas multi-environment model (3) incorporated a random effect ***f*** that attempts to explain a portion of the genotypic variance that is not explained by ***u*** and therefore has the potential to further capture that variability, which will improve prediction ability. The reaction norm model of Jarquín *et al.* (2014) applies this principle when adding a genomic component to the phenotypic component or adding ECs to explain environmental variability. We did not add any ECs to our model; therefore, matrix ***f*** will have a predictive effect only when lines are predicted in one environment using information from other correlated environments (random cross-validation scheme CV2). Under the random cross-validation scheme, where certain lines were not observed in any of the environments (cross-validation scheme CV1), there is no borrowing of information from the lines in the training set to predict other lines not observed in any of the environments (testing set); therefore, matrix ***f*** is not predictable and the prediction ability for one environment will be the same as that obtained by single-environment model (1) (López-Cruz *et al.* 2015).

Results from five data sets show that the increase in prediction ability of model (3) over model (2) is a function of the magnitude of the absolute values of the variance–covariance between environments $\left(F_{E}\right)$ and the method used (linear or nonlinear kernel). In general, the increases in prediction ability of model (3) over model (2) are with GBLUP because, as mentioned, model (2) explained only part of the genotypic variance; on the other hand, the increases in prediction ability of GK model (3) with respect to GK model (2) are smaller than those observed in the GBLUP because the nonlinear kernel with model (2) takes most of the variability and does not leave much variability to be explained by the covariance between environments $\left(F_{E}\right).$ Model (3) adds a random effect ***f*** representing part of the interaction between genetic factors environment that were not captured by ***u***; when used with the GK, part of the small cryptic variations represented by the small epistatic effect might be included in f.

### Comparing prediction ability of multi-environment model (3) with other multi-environment genomic G × E models in the literature

In this section, we compare results obtained in this study with multi-environment model (3) using methods GBLUP and GK with those obtained by other models and methods for the same data set and published in other articles (Burgueño *et al.* 2012; Cuevas *et al.* 2016; López-Cruz *et al.* 2015). It should be pointed out that this comparison of results is not completely objective because different random partitions and different numbers of partitions were performed in the different studies.

#### Wheat data set 1:

The first researchers to study the prediction ability of genomic G × E models were Burgueño *et al.* (2012) using multivariate mixed linear models for the variance–covariance matrix of the G × E by means of the parsimonious factor analytic (FA) structure and a diagonal matrix for the variance of error. Clearly, for E2 and E3, correlations computed by fitting the FA model were lower than those computed by GBLUP and GK model (3), which incorporates the random effect of ***f*** (Table 4). Environment 1, which is negatively correlated with all the other environments, was better predicted by FA (0.553) than by GK model (3) (0.543), while GK model (3) predicted E4 better (0.525) than FA (0.510).

On the other hand, Cuevas *et al.* (2016) used a GK based on the marker × environment interaction model of López-Cruz *et al.* (2015) and decomposed the interaction into two components: the main effects of markers across all the environments and the specific effects of markers in each environment. The reason for these differences is that the model of Cuevas *et al.* (2016) assumes positive correlations between environments; when there is a negative correlation between environments, its predictive capacity declines, as happened with the relatively low prediction ability of E1 (0.458). GK model (3) (and also GBLUP model (3)) is more flexible, admitting any correlation (positive, zero, or negative) between environments, and therefore predicted all four environments (E1–E4) better than the EB-G × E and FA models (Table 4).

#### Maize data set 2:

This data set was used by Cuevas *et al.* (2016) for comparing the GBLUP marker × environment interaction of López-Cruz *et al.* (2015) with the proposed GK marker × environment interaction. Moderate but consistent increases in prediction ability for each environment were achieved by GK model (3) over model EB-G × E (Table 4). Also, GK model (2) had consistently higher prediction ability than EB-G × E.

#### Wheat data sets 3–5:

These three data sets were used by López-Cruz *et al.* (2015) to fit the marker × environment interaction GBLUP-ME. GBLUP model (3) and GK model (3) showed consistently higher prediction ability than model GBLUP-ME, except in E4 of wheat data set 3, where GBLUP-ME had a prediction ability of 0.516, which was higher than the accuracy of GBLUP model (3) and GK model (3).

### Some limitations of the proposed models

In this study, we used five data sets with the same number of individuals in all the environments. This does not seem to be a limitation when the main idea is to predict the same number of individuals in all environments. However, when the total number of individuals is different in different environments, then the within-environment $K_{j}$ is different in each environment and the MTM software cannot be used directly. Fitting models (2)–(3) in this case is more complicated because, although it is possible to fit the models with the mode of the integrated likelihood, this requires much computing time. Fitting models for a large number of environments, even when the same number of lines are evaluated in each environment, also requires much computing time.

A possible solution for reducing computing time is to reduce the number of parameters to be estimated by assuming that matrices $U_{E},F_{E}$ are proportional to the phenotypic correlation, which does not seem unreasonable if the response data are standardized. However, more research on the use of this simplification is required to establish whether the prediction accuracies thus obtained are similar to those computed using the proposed estimation method.

### Conclusions

The Bayesian genomic G × E models described, implemented, and used in this study are novel and overcome some of the limitations imposed by previous genomic G × E models. Models (2) and (3) allow an arbitrary genetic covariance structure between environments, because an unstructured covariance matrix was used and its parameters were estimated from the data. These multi-environment models can be implemented using existing software for GS such as MTM. The cross-validation used 50 replicates and predicted lines in environments where they had not been observed using two sources of information: genomic relationships between lines and genetic information between environments. In all five data sets, models (2) and (3) had higher prediction accuracies than single-environment model (1) regardless of the genetic correlation between environments. In general, models (2) and (3) with the nonlinear GK had higher prediction accuracies for the lines unobserved in the environments than those obtained by the linear kernel (GBLUP) method. Under G × E interactions such as those found in the five data sets studied in this article, nonlinear GK models (2) and (3) performed very similarly and had higher prediction accuracies than linear GBLUP models (2) and (3). These models are clearly superior to single-environment genomic model (1) with GBLUP and GK, and their results are also superior to previous results from more restrictive marker × environment models. Prediction accuracies of models (2) and (3) with GK were higher than those obtained by other models and methods.

## APPENDIX A

### Example for fitting multi-environment model (3)

### Require MTM package (de los Campos and Grüneberg, 2016)

##### Require BGLR package (de los Campos and Pérez-Rodríguez, 2014)

##### Requiere function CV2 (López-Cruz *et al.*, 2015)

###Data

library(BGLR)

library(MCMCpack)

data(wheat)

X<-wheat.X

Y<-wheat.Y

X<-scale(X,center=TRUE,scale=TRUE)

Y<-scale(Y,center=TRUE,scale=TRUE)

env<-c(1,2,3,4); y<-Y[,env]

G<-X%*%t(X)/ncol(X)

In<-diag(1,nrow(Y),nrow(Y))

set.seed(12345)

##Simulation of missing data CV2, see López-Cruz *et al.* (2015)

yNA<-CV2(Y,env) # generation of CV2

### Fit model (3) with MTM (see de los Campos and Grüneberg, 2016)

fm <- MTM(

Y = yNA,

K = list(  list( K = G, COV = list( type = ′UN′, df0 = 4, S0 = diag(4) ) ), list(

K = In, COV = list(type = ′UN′, df0 = 4, S0 = diag(4)) )

),

resCov = list( type = ′DIAG′, S0 = rep(1, 4), df0 = rep(1, 4)

), nIter = 10000, burnIn = 1000, thin = 5, saveAt = ′ex1′

)

# ###Extracting the estimated parameters

YHatInt <- fm$YHat

##### Predictive correlation

test<-is.na(yNA)

tst1<-test[,1];tst2<-test[,2];tst3<-test[,3];tst4<-test[,4]

COR.PT1<-cor(YHatInt[tst1,1],y[tst1,1])

COR.PT2<-cor(YHatInt[tst2,2],y[tst2,2])

COR.PT3<-cor(YHatInt[tst3,3],y[tst3,3])

COR.PT4<-cor(YHatInt[tst4,4],y[tst4,4])

#### Extracting estimates of variance parameters

COR.RES<-fm$resCov$R # residual covariance matrix

COR.u<-fm$K[[1]]$G # genetic covariance matrix UE

COR.f<-fm$K[[2]]$G # genetic covariance matrix FE

## APPENDIX B

**Table B1:** Wheat data set 1

Env.	Covariance Matrix $U_{E}$ (Upper Triangular) and Correlation Matrix (Lower Triangular) for ***u***				Covariance Matrix $F_{E}$ (Upper Triangular) and Correlation Matrix (Lower Triangular) for ***f***				Variance–Covariance Matrix **Σ** for ***ε***			
	E1	E2	E3	E4	E1	E2	E3	E4	E1	E2	E3	E4
GBLUP												
E1	0.534	−0.123	−0.121	−0.235	0.302	0.074	−0.095	0.063	0.238	–	–	–
E2	−0.243	0.480	0.388	0.255	0.207	0.423	0.300	0.159	–	0.164	–	–
E3	−0.247	0.834	0.451	0.283	−0.256	0.682	0.457	0.114	–	–	0.177	–
E4	−0.483	0.552	0.632	0.444	0.236	0.503	0.347	0.236	–	–	–	0.379
GK												
E1	0.728	−0.159	−0.224	−0.219	0.200	0.118	−0.003	0.094	0.154	–	–	–
E2	−0.221	0.714	0.666	0.344	0.483	0.299	0.126	0.096	–	0.149	–	–
E3	−0.287	0.860	0.839	0.438	−0.015	0.499	0.213	0.003	–	–	0.163	–
E4	−0.311	0.493	0.579	0.683	0.460	0.384	0.014	0.209	–	–	–	0.220

Empirical phenotypic correlation between environments: E1 *vs.* E2 = −0.019; E1 *vs.* E3 = −0.19; E1 *vs.* E4 = −0.12; E2 *vs.* E3 = 0.661; E2 *vs.* E4 = 0.411; E3 *vs.* E4 = 0.388. Variance–covariance matrix (upper triangular) and correlation matrix (lower triangular) for random effects *u*, *f*, and variance matrix for random errors ***ε*** of multi-environment model (3) including four environments (E1–E4) for linear kernel GBLUP and nonlinear Gaussian kernel (GK). Pair-wise sample phenotypic correlations between environments are given above. Env., environment; GBLUP, genomic best linear unbiased predictors: GK, Gaussian kernel.

**Table B2:** Maize data set 2

Env.	Covariance Matrix $U_{E}$ (Upper Triangular) and Correlation Matrix (Lower Triangular) for ***u***			Covariance Matrix $F_{E}$ (Upper Triangular) and Correlation Matrix (Lower Triangular) for ***f***			Variance–Covariance Matrix **Σ** for ***ε***		
	1	2	3	1	2	3	1	2	3
GBLUP									
E1	0.442	0.275	0.117	0.268	0.101	0.094	0.221	–	–
E2	0.524	0.622	−0.001	0.415	0.221	0.025	–	0.226	0.000
E3	0.255	−0.002	0.475	0.369	0.108	0.242	–	–	0.288
GK									
E1	0.620	0.319	0.204	0.140	0.022	0.016	0.161	–	–
E2	0.468	0.748	0.030	0.167	0.124	0.015	–	0.147	0.000
E3	0.318	0.043	0.663	0.116	0.116	0.136	—	—	0.171

Empirical phenotypic correlation: Sample phenotypic correlations: E1vsE2 = 0.388; E1 *vs.* E3 = 0.262; E2 *vs.* E3 = 0.153. Variance–covariance matrix (upper triangular) and correlation matrix (lower triangular) for random effects *u*, *f*, and variance matrix for random errors ***ε*** of multi-environment model (3) including three environments (E1–E3) for linear kernel GBLUP and nonlinear Gaussian kernel (GK). Pair-wise sample phenotypic correlations between environments are given above. Env., environment; GBLUP, genomic best linear unbiased predictors: GK, Gaussian kernel.

**Table B3:** Wheat data set 3

Env.	Covariance Matrix $U_{E}$ (Upper Triangular) and Correlation Matrix (Lower Triangular) for ***u***				Covariance Matrix $F_{E}$ (Upper Triangular) and Correlation Matrix (Lower Triangular) for ***f***				Variance–Covariance Matrix **Σ** for ***ε***			
	1	2	3	4	1	2	3	4	1	2	3	4
GBLUP												
E1	0.403	0.368	0.129	0.169	0.254	0.086	0.087	0.017	0.281	–	–	–
E2	0.729	0.632	0.329	0.204	0.403	0.179	0.036	0.067	–	0.184	–	–
E3	0.273	0.555	0.556	0.128	0.362	0.178	0.228	0.033	–	–	0.322	–
E4	0.448	0.432	0.289	0.353	0.070	0.327	0.143	0.234	–	–	–	0.366
GK												
E1	0.693	0.453	0.175	0.191	0.132	−0.014	0.033	−0.023	0.145	–	–	–
E2	0.638	0.727	0.302	0.248	−0.122	0.099	0.004	0.012	–	0.123	–	–
E3	0.229	0.386	0.841	0.171	0.267	0.037	0.116	−0.004	–	–	0.126	–
E4	0.269	0.342	0.219	0.725	−0.168	0.101	−0.031	0.142	–	–	–	0.163

Empirical phenotypic correlation: E1 *vs.* E2 = 0.527; E1 *vs.* E3 = 0.253; E1 *vs.* E4 = 0.259; E2 *vs.* E3 = 0.340; E2 *vs.* E4 = 0.328; E3 *vs.* E4 = 0.220. Variance–covariance matrix (upper triangular) and correlation matrix (lower triangular) for random effects *u*, *f*, and variance matrix for random errors ***ε*** of multi-environment model (3) including four environments (E1–E4) for linear kernel GBLUP and nonlinear Gaussian kernel (GK). Pair-wise sample phenotypic correlations between environments are given above. Env., environment; GBLUP, genomic best linear unbiased predictors: GK, Gaussian kernel.

**Table B4:** Wheat data set 4

Env.	Covariance Matrix $U_{E}$ (Upper Triangular) and Correlation Matrix (Lower Triangular) for ***u***				Covariance Matrix $F_{E}$ (Upper Triangular) and Correlation Matrix (Lower Triangular) for ***f***				Variance–Covariance Matrix **Σ** for ***ε***			
	1	2	3	4	1	2	3	4	1	2	3	4
GBLUP												
E1	0.483	0.111	0.004	0.133	0.409	0.230	−0.055	0.215	0.175	–	–	–
E2	0.234	0.467	0.291	0.246	0.585	0.378	0.091	0.199	–	0.242	–	–
E3	0.008	0.560	0.578	0.292	−0.163	0.280	0.279	0.105	–	–	0.230	–
E4	0.304	0.572	0.610	0.396	0.541	0.521	0.320	0.386	–	–	–	0.243
GK												
E1	0.968	0.353	−0.042	0.378	0.142	0.084	−0.021	0.059	0.111	–	–	–
E2	0.395	0.826	0.417	0.476	0.502	0.197	−0.007	0.045	–	0.176	–	–
E3	−0.044	0.470	0.952	0.439	−0.170	−0.048	0.107	0.009	–	–	0.129	–
E4	0.429	0.584	0.502	0.803	0.383	0.248	0.067	0.167	–	–	–	0.192

Empirical phenotypic correlation: E1 *vs.* E2 = 0.342; E1 *vs.* E3 = −0.054; E1 *vs.* E4 = 0.311; E2 *vs.* E3 = 0.328; E2 *vs.* E4 = 0.414; E3 *vs.* E4 = 0.223. Variance–covariance matrix (upper triangular) and correlation matrix (lower triangular) for random effects *u*, *f*, and variance matrix for random errors ***ε*** of multi-environment model (3) including four environments (E1–E4) for linear kernel GBLUP and nonlinear Gaussian kernel (GK). Pair-wise sample phenotypic correlations between environments are above. Env., environment; GBLUP, genomic best linear unbiased predictors: GK, Gaussian kernel.

**Table B5:** Wheat data set 5

Env.	Covariance Matrix $U_{E}$ (Upper Triangular) and Correlation Matrix (Lower Triangular) for ***u***					Covariance Matrix $F_{E}$ (Upper Triangular) and Correlation Matrix (Lower Triangular) for ***f***					Variance–Covariance Matrix **Σ** for ***ε***				
	1	2	3	4	5	1	2	3	4	5	1	2	3	4	5
GBLUP															
E1	0.626	0.161	0.186	−0.039	0.017	0.141	0.035	0.010	−0.016	0.008	0.193	–	–	–	–
E2	0.277	0.538	−0.022	0.092	0.030	0.227	0.169	0.006	−0.004	0.016	–	0.277	–	–	–
E3	0.301	−0.038	0.608	−0.099	−0.018	0.078	0.043	0.116	0.037	0.061	–	–	0.140	–	–
E4	−0.063	0.161	−0.163	0.605	0.405	−0.086	−0.020	0.219	0.247	0.180	–	–	–	0.189	–
E5	0.029	0.055	−0.031	0.705	0.546	0.038	0.070	0.323	0.653	0.308	–	–	–	–	0.201
GK															
E1	0.849	0.201	0.261	−0.094	0.010	0.083	0.014	0.003	−0.009	0.005	0.100	–	–	–	–
E2	0.232	0.887	0.014	0.095	0.032	0.150	0.105	0.006	−0.006	0.013	–	0.136	–	–	–
E3	0.325	0.017	0.759	−0.031	0.060	0.040	0.071	0.068	0.012	0.014	–	–	0.082	–	–
E4	−0.105	0.104	−0.037	0.949	0.677	−0.095	−0.056	0.139	0.109	0.031	–	–	–	0.135	–
E5	0.011	0.035	0.072	0.722	0.927	0.047	0.108	0.145	0.254	0.137	–	–	–	–	0.174

Empirical phenotypic correlation: E1 *vs.* E2 = 0.166; E1 *vs.* E3 = 0.30; E1 *vs.* E4 = −0.10; E1 *vs.* E5 = −0.010; E2 *vs.* E3 = −0.033; E2 *vs.* E4 = 0.122; E2 *vs.* E5 = 0.035; E3 *vs.* E4 = −0.091; E3 *vs.* E5 = 0.023; E4 *vs.* E5 = 0.546. Variance–covariance matrix (upper triangular) and correlation matrix (lower triangular) for random effects *u*, *f*, and variance matrix for random errors ***ε*** of multi-environment model (3) including five environments (E1–E5) for linear kernel GBLUP and nonlinear Gaussian kernel (GK). Pair-wise sample phenotypic correlations between environments are given above. Env., environment; GBLUP, genomic best linear unbiased predictors: GK, Gaussian kernel.
